# Deep learning methods for the detection of stroke for neuropathology: Lessons learned from *in vivo* imaging to digital pathology

**DOI:** 10.1017/cts.2026.10799

**Published:** 2026-07-28

**Authors:** Joohi Chauhan, Audrey P. Fan, Shino D. Magaki, Amparo C. Villablanca, Harry V. Vinters, Sen-ching S. Cheung, Chen-Nee Chuah, Brittany N. Dugger

**Affiliations:** 1 Department of Electrical and Computer Engineering, University of California Davis, Davis, CA, USA; 2 Computer Science and Engineering, Motilal Nehru National Institute of Technology Allahabad, Prayagraj, UP, India; 3 Department of Biomedical Engineering, University of California Davis, Davis, CA, USA; 4 Department of Pathology and Laboratory Medicine, University of California Los Angeles, Los Angeles, CA, USA; 5 Department of Internal Medicine, University of California Davis, Davis, CA, USA; 6 Electrical and Computer Engineering, University of Kentucky, Lexington, KY, USA; 7 Department of Pathology and Laboratory Medicine, University of California Davis, Davis, CA, USA

**Keywords:** Stroke, cerebral infarcts, machine learning, *in vivo* imaging, digital pathology

## Abstract

Stroke is a leading cause of death and a major contributor to long-term disability worldwide. Accurate stroke assessment is critical for both research and clinical care. *In vivo* neuroimaging modalities, particularly CT and MRI, are widely used and increasingly coupled with deep learning (DL) to improve stroke assessment. These approaches have matured rapidly and benefit from harmonized imaging protocols and growing multi-center datasets, but they remain limited in sensitivity to microinfarcts, subtle early ischemic changes, and cellular-level pathology. Digital pathology, through whole-slide imaging, offers unmatched tissue-level resolution and biological ground truth for validating imaging biomarkers, yet DL applications for infarct analysis in histopathology remain comparatively underdeveloped and data-limited, with most existing work focused on vascular morphometry or neurodegenerative pathology rather than infarct analysis. This review aims to provide insights from pathology, radiology, and computational sciences. We summarize dominant architectural trends in *in vivo* imaging, review emerging computational pathology approaches, and describe MRI-pathology correlation and *ex vivo* validation workflows. We further examine key translational challenges, including dataset scarcity, scanner and staining variability, demographic bias, computational feasibility, and limited external validation. Finally, we outline future directions emphasizing harmonized data infrastructure, multimodal integration, and emerging foundation models to enable scalable, biologically grounded, and equitable infarct assessment across clinical and research settings.

## Introduction

### Epidemiology and burden of stroke and diagnostic challenges

Stroke is a major global health concern, the second leading cause of death, and a leading cause of morbidity and disability worldwide. Globally, the prevalence of stroke (currently 15 million) is increasing, particularly in middle to low-income communities, as is its contribution to vascular dementia [1]. Globally, the lifetime risk of stroke (for age 25 onwards) is estimated to be 25.1% for women and 24.7% for men, with significant regional variations [2]. Stroke is the fifth leading cause of death overall in the United States (US), the fourth leading cause of death among women, and the third leading cause of death among both African American and Hispanic women [2–4]. Moreover, the economic burden of stroke is projected to rise by 129% from 2013 to 2030, with an increase in the direct financial burden of stroke-related medical costs from $71.6 billion in 2013 to $184.1 billion in 2030 [5].

While precise assessment of stroke is essential for timely diagnosis and intervention, current approaches often lack consistency, scalability, and generalizability across populations. Given the high disease burden of stroke, it remains of high importance for timely and accurate diagnosis to facilitate prognosis and treatment. This review examines how emerging technologies, such as machine learning (ML), have aided in the evaluation/assessment of stroke in *in vivo* imaging and how leveraging these concepts and adapting to pathology may improve understanding, diagnostics, and clinical outcomes. As such, we review progress made in *in vivo* imaging and how it may aid in pathology assessments to (1) foster innovation (by leveraging ML technologies and methodologies to address translational gaps), (2) enhance efficiency in the translational pipeline, and (3) discuss implementation challenges including multi-scale MRI-histology registration, tissue deformation during histologic processing, domain adaptation across imaging modalities, dataset limitations, and external validation.

### An evolving understanding of cerebral infarcts, the pathologic manifestation of stroke

Clinically, stroke is defined as an acute cerebrovascular syndrome resulting from either interruption of cerebral blood flow (ischemic stroke) or intracranial hemorrhage (hemorrhagic stroke) [8]. In contrast, a cerebral infarct is a pathologic entity referring specifically to the region of irreversible tissue necrosis caused by prolonged ischemia [6]. Accurate identification and characterization of infarcts are essential for diagnosis, treatment selection, and outcome prediction.

Cerebral infarcts are biologically heterogeneous and evolve dynamically over time, with changes in tissue structure, cellular composition, and vascular integrity [9,16,18]. This temporal and spatial variability contributes to challenges in infarct detection and classification across both *in vivo* imaging and neuropathological evaluation [21,24,57]. In particular, the limited spatial resolution and contrast sensitivity of CT and MRI reduce sensitivity for microinfarcts, small chronic lesions, and subtle tissue remodeling, leading to underestimation of true infarct burden and incomplete biological characterization [21,22,24]. Although advances in imaging and pathology have improved understanding of infarct biology, substantial inter-observer variability persists, particularly for small or chronic lesions and for estimating infarct age [13,19,20]. These challenges present opportunities for ML to enable harmonized, data-driven approaches to aid in assessing the full spectrum of infarct pathology.

### Categorization of cerebral infarcts

Neuropathological evaluation remains the gold standard for identifying ischemic necrosis and classifying cerebral infarcts based on size, age, anatomical location, and the presence or absence of hemorrhagic components [17]. At a high level, infarcts are commonly categorized by size (large/cystic, lacunar, or microinfarct), temporal stage (acute, subacute, or chronic), and underlying mechanism (ischemic and/or hemorrhagic), shown in Figure 1 (additional details in Supplemental Table A1, and Figures A1 and A2). These classification frameworks provide an important conceptual foundation for clinical and research studies.

**Figure 1.**
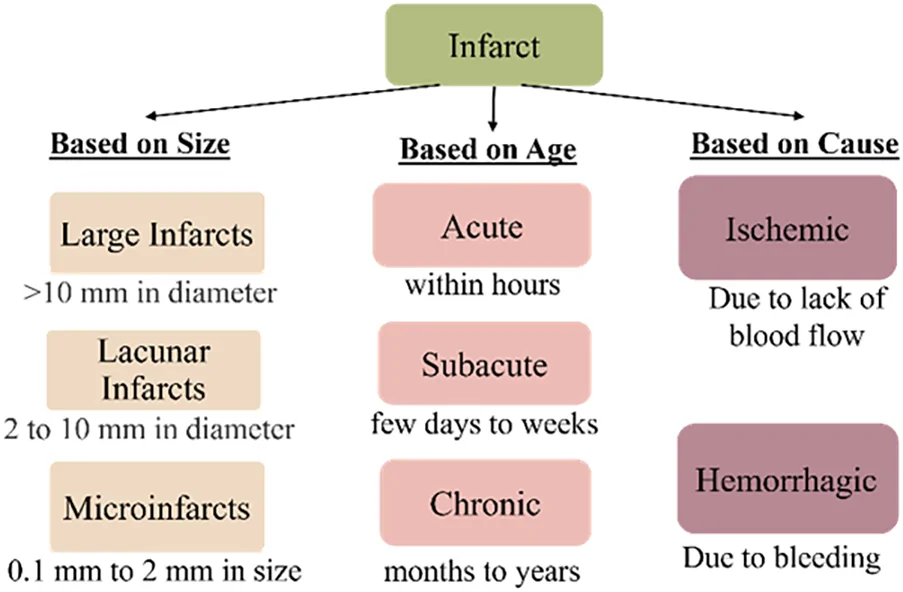
Brief overview of categorization of cerebral infarct. Based on the size, an infarct can be classified as a large (>10 mm), lacunar (2–10 mm), or microinfarct (lesions not visible by gross inspection of the brain but seen through microscopic evaluation) [13,14]. In the age category of infarct, acute infarcts typically occur within hours before a person’s death, subacute infarcts generally happen within a few days to weeks, and chronic infarcts usually are months to years old [15,16]. However, the “dating/timing” of brain infarcts based on gross and microscopic features may have differential definitions (see Table A1 in the supplementary material for additional details/summary of studies). The last category is based on cause, ischemic and/or hemorrhagic, which may have a simultaneous onset. A hemorrhagic infarct reflects bleeding into an area of necrosis.

Microinfarcts present a particular diagnostic challenge, as they are not visible on gross examination and fall below the resolution of conventional *in vivo* neuroimaging. These lesions are typically submillimeter in size, with only the largest acute microinfarcts occasionally visible on diffusion-weighted imaging [21].

### Literature search strategy

To aid in providing a synopsis of recent advances in ML and DL for stroke analysis across *in vivo* imaging and digital pathology, we conducted a brief literature review. Peer-reviewed articles on *in vivo* studies were identified through searches of PubMed, Scopus, and Web of Science, performed between February 2023 and September 2025, targeting publications from 2020 to 2025. Search terms included combinations of keyword-based queries related to “stroke,” “cerebral infarct,” “deep learning,” “machine learning,” “CT,” “MRI,” “digital pathology,” and “whole slide imaging.” Studies were included if they applied ML/DL methods to stroke or infarct detection, segmentation, classification, or outcome prediction and reported quantitative performance metrics on humans. Studies were excluded if they were not based on humans, did not offer primary methodological contributions, addressed tasks unrelated to infarct analysis, or were published in languages other than English.

Studies were selected to represent key methodological developments across years (2020–2025), task types, and imaging modalities, rather than to provide an exhaustive or systematic listing.

### *In Vivo* imaging of cerebral infarcts

*In vivo* neuroimaging forms the clinical foundation for the diagnosis and management of cerebral infarction. A range of imaging modalities (see supplementary material Table A2) is used in routine clinical practice to detect stroke, characterize lesion extent, and guide treatment decisions. However, intrinsic limitations in spatial resolution, contrast sensitivity, and interpretive variability motivate ongoing efforts to enhance infarct detection and characterization using computational and ML-based approaches.

## Relationships of in vivo imaging to pathology

The biological features of infarcts vary by vascular territory, infarct size, and temporal stage, contributing to diverse imaging appearances and clinical outcomes [16,19,22]. There are numerous published studies as well as guidelines that have aided in providing guidance on aligning *in vivo* neuroimaging findings with those found pathologically, focusing on segmentation, registration, and interpretation of imaging in the context of tissue pathology [9,23,25,95–98]. One of the founding papers, published in 1996 (reviewed in [107]), provided a workflow for correlating lobar cerebral infarction postmortem MRI features with both gross and histologic pathology, as well as *in vivo* neuroimaging. Building on this framework, subsequent *ex vivo* and ultra-high-field postmortem MRI studies demonstrate improved MRI-histology alignment, supported by structured *ex vivo* MRI-pathology workflows (Figure 2), and reveal imaging-visible lesions often reflect heterogeneous microscopic pathology not fully captured by *in vivo* imaging [95,96]. Targeted sampling workflows using three-dimensional cutting guides have further improved spatial accuracy and reproducibility [98]. A small but important set of studies has demonstrated histopathology provides essential biological validation for imaging-defined cerebrovascular lesions. Post-mortem MRI-guided pathology investigations show imaging abnormalities frequently correspond to heterogeneous microscopic substrates rather than a single uniform pathological process [95]. Similarly, Lahna et al. used MRI-guided histological sampling to characterize white-matter hyperintensities and microvascular injury, highlighting histopathology as a critical ground truth for interpreting imaging biomarkers [96]. Standardized *ex vivo* MRI workflows further preserve anatomical fidelity and reduce deformation artifacts during tissue processing [97]. Collectively, these studies underscore the indispensable role of histopathology in validating and contextualizing imaging-derived stroke biomarkers, while also highlighting the scarcity of large, standardized MRI-pathology cohorts. Figure 2 provides a schematic of an example MRI-pathology registration workflow [95–97,118–121]. The resulting spatially registered histology datasets could aid in providing the experimental foundation for subsequent computational pathology and ML-based infarct analysis pipelines.

**Figure 2.**
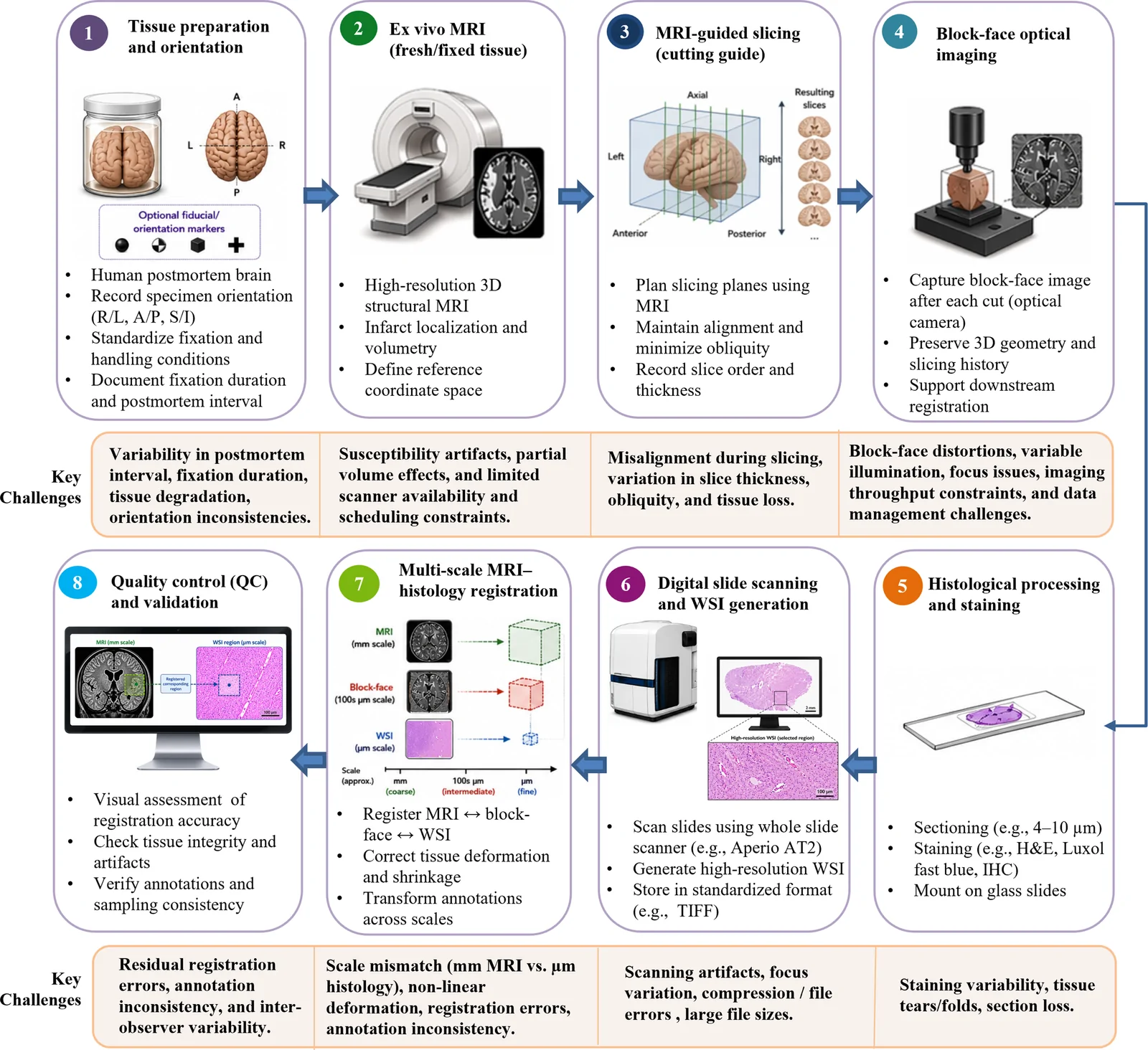
Potential 8 step workflow (box 1 upper left to box 8 lower left) for MRI-pathology registration and whole-slide image (WSI) generation in cerebral infarct analysis, highlighting key processing stages, multi-scale registration, and major technical challenges across the pipeline [95–97, 118–121]. *Disclosure: The illustrative images used in each step are generated using an AI image generator tool [114] and are subsequently modified, assembled, and refined by the authors to create the final conceptual workflow schematic. The figure is intended as a representative conceptual illustration synthesized from published MRI-histopathology studies and does not depict a single experimental protocol or dataset.*

Despite the clinical utility, *in vivo* imaging modalities (CT, MRI, PET) remain constrained in their ability to detect microinfarcts, resolve cellular-level pathology, and accurately determine infarct timing [12,25–27]. As summarized in Table A2 (supplementary material), each modality involves trade-offs between speed, sensitivity, resolution, and accessibility. Consequently, multimodal imaging is often required for comprehensive stroke evaluation. While detailed histopathological features such as macrophage infiltration, reactive gliosis, and tissue cavitation provide critical biological insight, these microscopic changes are often incompletely captured by *in vivo* imaging [21]. These modality-specific limitations (particularly limited sensitivity to subtle lesions, variability across scanners and protocols, and reliance on expert interpretation), together with the inherent biological heterogeneity of infarcts, motivate the development of computational approaches that can integrate multi-scale information, reduce observer-dependent variability, and extract quantitative biomarkers from complex imaging data.

## Use of machine learning for in vivo imaging and detection of cerebral infarcts

Artificial intelligence (AI) encompasses computational methods that enable systems to learn from data and perform perception, prediction, and reasoning tasks that support or extend human decision-making [28]. Figure 3 illustrates the different subtypes of AI and their relationships. Within this domain, ML and its subset deep learning (DL) have become central to medical image analysis, providing automated, data-driven solutions for detecting and characterizing strokes from radiological data [29,30].

**Figure 3.**
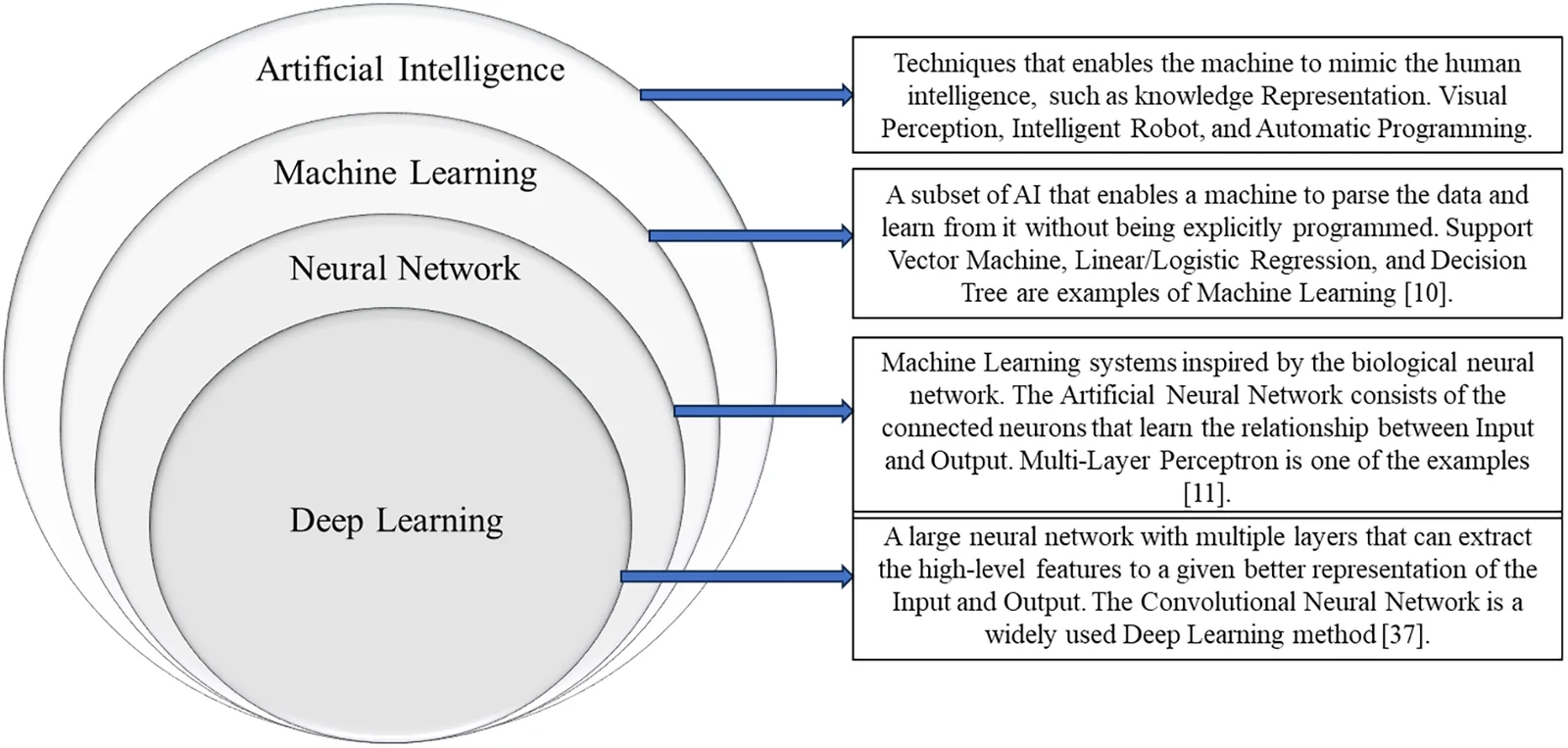
Artificial Intelligence (AI) and the relationship between its subtypes.

In *in vivo* stroke assessment, ML/DL frameworks have enhanced diagnostic accuracy, assisted triage, and enabled quantitative biomarkers that link imaging patterns to underlying pathophysiology and outcomes [32,46,78,81]. When applied to CT and MRI, ML models improve sensitivity and specificity compared with conventional assessment, offering automated diagnostic support for classification and segmentation tasks [28–33]. Annotated images serve as labeled data or ground truth for model training, forming the basis for computer-aided detection and diagnosis algorithms that accelerate radiologic workflows [86–90].

Early ML studies for *in vivo* stroke detection relied on handcrafted radiomic and perfusion features classified by algorithms such as support vector machines, random forests, or gradient boosting [76]. The field has since shifted toward DL architectures that learn hierarchical representations directly from image data, enabling automated feature extraction, improved robustness to imaging variability, and superior scalability compared with manual feature engineering [37,41]. Convolutional neural networks (CNNs) and encoder-decoder structures such as U-Net remain the dominant backbones for *in vivo* imaging stroke lesion segmentation, as U-Net captures fine spatial details while aggregating multiscale contextual information [37,41,73,81,84]. Although U-Net substantially improved lesion delineation accuracy, challenges remain for rare pathology/classification subtypes, small lesions, class imbalance, and limited availability of well-annotated clinical imaging datasets [39,40,46,63]. Consequently, transfer learning approaches on CNN were utilized after the ImageNet Large Scale Visual Recognition Challenge in 2012 [99], and transfer learning leveraged pre-trained feature representations from large-scale medical or natural image datasets [36]. Federated learning and domain adaptation methods introduced in 2017 have more recently been explored to enable multi-institutional model development without centralized data sharing, addressing regulatory, ethical, and patient privacy constraints that limit cross-site data aggregation [38,100,101].

Building on this foundation, more recent research has explored architectural extensions including residual connections, multi-scale feature pyramids, attention mechanisms, and CNN-transformer hybrids (e.g., Swin-Transformer and SegFormer-style models) to better capture global context and improve robustness to lesion heterogeneity and low-contrast ischemic changes, particularly on non-contrast CT [41–43,73,81,84,87,89]. However, across multiple studies, performance differences attributable to architectural choice are often smaller than those driven by dataset size, imaging modality, and cohort composition.

Table 1 highlights some of these developments as representative methodological milestones from the research literature (2020–2025), illustrating both the dominance of U-Net-based encoder-decoder frameworks and the incremental gains achieved through architectural refinements. Importantly, the table also highlights a persistent limitation of the field: architectural diversity has outpaced diversity in dataset composition, cohort size, and validation strategies, constraining cross-study comparability and generalizability.

**Table 1.** Summary of selected DL/ML milestone methods employed for *in vivo* brain stroke and infarct analysis, grouped by primary task category, publication year, and imaging modality Table 1 long description.

Paper	Imaging modality	Method	Datasets description	Performance	Main advancement / contribution
**Segmentation methods**					
Wang et al., 2025 [89]	NCCT	*Infarct lesion segmentation:* VB-Net (dual-channel); DA-Net for ASPECTS region extraction. Classification: reperfused and non-reperfused; K-best algorithm Prediction: Infarct Volume; Gradient boosting decision tree.	Images: 397 [Training: 345, Testing: 52] (AISD) Classification Images: 276 [Training: 220, Validation: 56] Demographic information not reported	Segmentation DC: 76%, Classification AUC: 0.837 R: 83.3%	Integration of infarct segmentation with reperfusion and outcome prediction.
Ince et al., 2025 [81]	MRI Diffusion-Weighted Imaging	*Segmentation of Cerebral Vascular Occlusion:* U-Net + ConvNeXtV2 blocks + GRN-based MLP.	400 individuals [Training: 250, Testing: 150]; Image resolution: 256x256 pixels; demographic information not reported	IoU: 80.15%, DC: 88.94%.	Modern CNN–transformer hybrid achieving high Dice with improved feature hierarchy.
Jazzar & Douik, 2025 [84]	MRI	*Ischemic stroke lesion segmentation*: Res2U++ (hybrid ResUnet++ encoder and modified U-Net decoder); ASPP block.	ISLES 2022: 400 individuals [Training: 250, Testing: 150]; SISS 2015: 64 individuals [Training: 28, Testing: 36]; demographic information not reported	ISLES 2022: DC: 85%, Acc: 98% SISS 2015: DC: 85%, Acc: 98.6%.	Hybrid encoder–decoder refinement improving feature reuse and multi-scale representation.
Liu et al., 2023 [41]	MRI	*3D Segmentation-stroke lesion and Thrombolysis in Cerebral Infarction) grading:* Residual U-net, MLP, and SQM simulated quantum mechanics)	655 individuals [Training: 524, Testing: 131]; demographic information not reported	DC: 71% Acc: 86.78%	Channel attention for feature weighting and boundary refinement in infarct segmentation.
Xu et al., 2023 [42]	CT	*Segmentation-stroke lesion:* CNN with attention Gate and ASPP	203 individuals (149 male and 54 female). 5998 cranial CT slices (3941 normal and 2057 hemorrhage); Image resolution: 512 × 512	mIoU: 85.3% DC: 91.8%	Introduced attention mechanisms and multi-scale feature extraction to improve detection of subtle NCCT lesions.
Yu et al., 2020 [46]	Perfusion-weighted Imaging and Diffusion-Weighted Imaging	*Segmentation-ischemic stroke lesions:* FCN with Adam optimizer and different Loss functions, such as Weighted Binary Cross-Entropy, L1, and Dice Loss	182 individuals (97 female and 85 male) average age 65 years. [5-fold cross-validation]	Sen: 66.0% DC: 53%	Early multimodal input fusion for infarct estimation; exposed challenges in modality alignment and robustness.
Zhang et al., 2020 [45]	Diffusion-Weighted Imaging	*Segmentation-acute ischemic lesions:* FCN with SGD optimizer and MSE and Cross Entropy Loss	28 individuals [Training: 21 (3213 images), Testing: 7 (1071 images)]; Image resolution: 230 × 230 × 153 voxels; demographic information not reported.	P: 35% R: 43% DC:58%	Demonstrated FCN applicability for small datasets; highlighted sensitivity to limited training data and noise.
Hu et al., 2020 [44]	MR Perfusion	*Segmentation of brain stroke lesion:* CNN with Adam optimizer and Focal loss	75 individuals [Training: 43, Testing: 32] Image resolution: 96 dpi; demographic information not reported	DC: 30%	Early fully convolutional baseline demonstrating feasibility of automated infarct segmentation; limited accuracy and generalization.
**Detection methods**					
Wang et al., 2025 [86]	CT, MRI	*Ischemic stroke lesion detection:* ISFPN (Feature Pyramid Network + Swin Transformer).	ISLES2018-CT: 103 individuals [Training: 63, Testing: 40] ISLES2022-MRI: 400 individuals [Training: 250, Testing: 150]; demographic information not reported	ISLES2018-CT: P: 45.72%, R: 38.45%, ISLES2022-MRI: P: 79.09%, R: 54.22%.	First transformer-based multi-scale detection framework modeling global spatial context.
Dong et al., 2026 [103]	3D CTA	Multi acute stroke task: Image encoder: ViT, DeepSymNetv3, ResNet18 Text Encoder: OpenCLIP, DistilBERT, BioClinicalBERT	1542 individuals (685 male and 857 female)	LVO: AUC: 0.85 AIS vs. ICH: 0.95	Introduced self-supervised contrastive pretraining using paired CTA and radiology reports to improve generalization across multiple acute stroke tasks.
Abumihsan et al., 2024 [87]	MRI	*Ischemic stroke detection:* Convolutional Block Attention Module.	Images: 2000 [Training: 1,600, Testing: 400]; Image resolution: 224 × 224 pixels; demographic information not reported	Acc: 98.75%, P: 98.80%, R: 98.70%, F1: 98.75%, AUC:99.1%	Demonstrated attention modules improve robustness and scalability on larger image datasets.
Negm et al., 2023 [113]	CT	*Intracranial Hemorrhage Diagnosis, detection, and classification,* the MVEDL technique is used, which comprises ELM-SAE, RNN, and BiLSTM models.	36 individuals [Training: 273, Testing: 68]; Demographic information not reported	Acc: 97.10% F1: 89.21% P: 96.18%	The study presents an Intracranial Hemorrhage Diagnosis using Willow Catkin Optimization with Voting Ensemble Model on CT images.
**Classification/Outcome prediction methods**					
Koska et al., 2025 [85]	MRI Diffusion-Weighted Imaging	*Patient-level classification of affected vascular territory in ischemic stroke*: End-to-end deep learning (CNN-based) model; multi-class classification (MCA, PCA, ACA, vertebrobasilar)	1052 individuals [Training: 841, Validation: 105, Testing: 106]; demographic information not reported	Acc: 93.4% F1: 93.2%	Developed end-to-end deep learning models performed well in classifying affected regions from stroke in DWI.
Yang et al., 2024 [78]	MRI	*Prediction of 3-month functional outcomes after acute ischemic stroke*: ResNet50 iterative attentional feature fusion.	3338 individuals (2018 male and 1320 female) [Training: 2018, Validation: 660, Testing: 660]	AUC: 0.788	Large cohort modeling for functional outcome prediction beyond lesion segmentation.
Ferdous & Shahriyar, 2024 [80]	CT	*Stroke Prediction*: Ensemble CNN model ENSNET, Inceptionv3, MobileNetv2, and Xception; Transfer learning.	Images: 2515 [Training: 1843 (1087 normal, 756 stroke), Validation: 235 (157 normal, 78 stroke), Testing: 437 (307 normal, 130 stroke)]; Image resolution:224 × 224; demographic information not reported.	Acc: 98.86%, P: 97.71%, R: 98.46%, F1: 98.08%, AUC:98.7%	Propose a new ensemble model to predict brain strokes. Also, uses analytics assessment metrics to validate the performance of the suggested ensemble model.
Bridge et al., 2022 [32]	MRI Diffusion-Weighted Imaging	*Classification* (Infarct, Normal) *Segmentation:* U-Net with Adam optimizer and Dice and Binary cross-entropy loss	Images for classification: 8174 [Training: 6657, Validation: 725, Testing: 792]; images for segmentation: 473 [Training: 377, Validation: 34, Testing: 62]. demographic information not reported.	AUC: 99.8% Sen.: 98.4% Spec.: 98% DC: 81.3%	Established encoder–decoder CNN dominance for infarct segmentation with substantial performance gains over FCNs.
Gautam et al.,2021 [31]	CT	*Classification:* (Stroke: ischemic, hemorrhagic, normal) CNN	74 individuals (45 male and 29 female); Images: 900 [Training: 720, Testing: 180]; Image resolution: 512 × 512; demographic information not reported	Acc.: 92.2% P: 92% R: 92%	Early CNN-based stroke classification on NCCT demonstrated a transition from traditional ML toward DL but was limited by a small cohort size and a lack of segmentation capability.

(Acc = Accuracy, P = Precision, R = Recall, Spec = Specificity, Sen = Sensitivity, DC = Dice similarity coefficient, SSIM = structural similarity index measure, RC = Roger-Tanimoto Coefficient, SC = Sokal-Michener Coefficient, SS = Sokel-Sneath-2, RI = Russell-Rao Index, SI = Sorenson Index, MCC = Mathew’s Correlation Coefficient, ICH = intracerebral hemorrhages, AIS = acute ischemic stroke, LVO = Large vessel occlusion). Blue-shaded rows indicate segmentation methods, peach-shaded rows indicate detection methods, and green-shaded rows indicate outcome prediction methods.

Parallel to these research advances, a subset of AI algorithms for stroke imaging has achieved FDA clearance and demonstrated practical diagnostic and workflow benefits in acute cerebrovascular care [105,106,109–112]. Systematic evaluations, including a comprehensive review by Al-Janabi et al., report that clinically deployed platforms such as RapidAI, Brainomix®, and Viz.ai achieve high sensitivity and specificity for intracranial hemorrhage, acute ischemic stroke, and large-vessel occlusion detection, enabling faster triage and decision-making compared with conventional interpretation alone [110]. For example, the Rapid NCCT Stroke platform (FDA 510(k) K222884) demonstrated very high performance for intracranial hemorrhage detection (∼96.2% sensitivity, ∼99.5% specificity) and moderate sensitivity for large-vessel occlusion detection (∼63.5%) in multicenter retrospective cohorts, outperforming general radiologists for selected tasks on non-contrast CT [105,106,109].

Collectively, while Table 1 captures the evolution of DL architectures in research settings, FDA-cleared stroke AI systems may represent a downstream translational subset that prioritizes robustness, speed, and workflow integration over incremental architectural complexity.

Beginning around 2020, transformer-based and hybrid CNN-transformer architectures have been explored to capture long-range spatial dependencies and global context, improving segmentation (in terms of dice coefficient) of large territorial infarcts and diffuse ischemic changes [78–81,84,86,89]. Architectures such as Vision Transformers, Swin Transformers, and hybrid attention-augmented U-Nets incorporate self-attention mechanisms that model spatial relationships across distant regions of the brain [41,42,73,78,81,86]. Across multiple studies, performance differences attributable to architectural choice (e.g., U-Net derivatives versus hybrid CNN or transformer backbones) are often smaller than those driven by dataset size, modality, and cohort composition. For example, ischemic stroke lesion detection based on Swin Transformer backbone achieves precision and recall of 46% and 38%, respectively, for the CT dataset with 103 cohort size, whereas the same model achieves precision and recall of 79% and 54%, respectively, for the MRI dataset with 400 cohort size [86]. Recent studies, one based on DenseNet121 encoder and SelfONNCSCA [90], and the other based on Res2U++ (modified U-Net) [84], evaluated on the same dataset, ISLES 2022-MRI [116] with a cohort size of 400, achieved dice coefficient 86% and 85%, respectively. These findings suggest dataset size and modality characteristics exert greater influence on performance than incremental architectural complexity.

ML model performance can also vary substantially by imaging modality. In non-contrast CT, early ischemic changes are subtle and low contrast, motivating ML architectures that employ multi-scale feature extraction and residual learning (such as ResNet backbones, feature pyramid networks, Atrous Spatial Pyramid Pooling (ASPP), and multi-scale U-Net variants) to amplify weak intensity differences between infarcted and normal tissue [78,81,84,86]. In diffusion-weighted MRI (DWI), lesions appear hyperintense with clearer boundaries, enabling CNN-based U-Net derivatives to achieve consistently higher Dice coefficients [86,90]. Multimodal fusion approaches combining NCCT with CTA, CT perfusion, or multi-sequence MRI further reflect clinical reasoning by integrating vascular status, tissue viability, and diffusion abnormalities, and often improve sensitivity for penumbra and infarct estimation [88,89]. However, these approaches can increase computational complexity and depend on complete data availability [41,46].

Quantitatively, segmentation Dice coefficients in the most recent (2025) MRI-based studies [81,84,90] typically range from 0.839 to 0.890, while the NCCT-based model VB-Net (dual-channel) [89] achieves a Dice coefficient of 0.760 depending on lesion visibility. In contrast, earlier infarct segmentation approaches (2020), primarily based on fully convolutional networks (FCNs) and shallow CNN architectures, reported substantially lower Dice coefficients, commonly in the range of 0.30–0.58, particularly for early DWI MRI datasets [44–46]. These earlier models were limited by small training cohorts (typically 21–182) and handcrafted or shallow feature representations, resulting in reduced robustness to imaging variability. These trends underscore reported Dice or AUC values often reflect dataset-specific optimization rather than true generalizable performance [40]. Direct comparison of performance metrics across studies should be interpreted with caution. Reported values such as Dice coefficient or AUC can be influenced by multiple factors beyond model architecture, including imaging modality (e.g., CT vs MRI), lesion characteristics (e.g., size, location, and stage), dataset size and composition, preprocessing pipelines, and validation strategies. Differences in annotation protocols and inter-rater variability may further contribute to variability in reported performance. As a result, apparent differences in model performance across studies often reflect variations in dataset quality, task definition, and evaluation design, rather than inherent superiority of specific architectures. Accordingly, these metrics should be viewed as study-specific indicators rather than directly comparable benchmarks.

Despite encouraging performance metrics, reproducibility and generalizability remain major obstacles [33,40,91]. Many published models are trained on single-center, retrospective datasets that differ in scanner type, acquisition parameters, preprocessing, and annotation standards [36,39,61]. Public datasets such as ISLES [116], ATLAS [117], and SPES [116] have provided valuable benchmarks but remain limited in cohort diversity, lesion heterogeneity, and imaging protocols, making external validation essential. Domain shifts between 1.5 T, 3 T, and 7 T MRI systems or between CT reconstruction protocols lead to degraded cross-site performance [96]. Inter-rater variability can introduce label noise that limits achievable accuracy, and few studies conduct external validation or prospective testing [63,102].

DL-based *in vivo* stroke imaging has matured from handcrafted ML approaches to sophisticated hybrid and multimodal architectures that approach expert-level performance for many segmentation tasks. Nevertheless, intrinsic limits in spatial resolution, coupled with persistent challenges in data diversity and reproducibility, restrict the ability of CT and MRI to characterize microinfarcts and cellular-level pathology [33,59] even with the implementation of ML algorithms. These limitations motivate the exploration of histopathological and digital pathology-based approaches, which offer complementary insight into infarct biology at microstructural and molecular scales, as discussed in the following section.

## Machine learning for digital pathology and infarct detection

Digital pathology has emerged as a transformative approach for quantitative characterization of cerebrovascular lesions. It enables the analysis of high-resolution histological whole slide images (WSIs) generated by scanning tissue slides with specialized scanners and provides microscopic validation of neuroimaging findings and can be paired with imaging software, such as QuPath [47,48]. WSIs provide a detailed digital version of morphological, cellular, and architectural features, preserving spatial context crucial for pathologic diagnoses [47]. These rich data enable the training of DL models for tasks such as classification, segmentation, and/or disease detection [49,92]. While such approaches have been widely applied in other tissue types and diseases, they have begun to emerge in neurodegenerative diseases [53,75,94,102], their application to cerebral infarcts remains comparatively underdeveloped. Cerebral infarcts represent dynamic ischemic injury with heterogeneous spatial and temporal features, requiring task definitions specifically aligned with infarct biology, although numerous guidelines exist, there is high inter-rater reliability, especially for microinfarcts, and there is not one universal accepted criterion for the diagnosis of cerebral vascular entities [13,20], as discussed above.

Existing infarct-focused pathology studies remain limited in cohort size and task scope, highlighting substantial opportunities for scalable, data-driven characterization of ischemic and hemorrhagic tissue microstructure [92]. Related work in anatomic segmentation or pathology classification models have overlap with ischemic tasks but are not typically framed as cerebral infarct analysis [50,94,102]. As detailed in Section above, MRI-pathology correlation studies provide the biological foundation for these computational approaches.

To address this gap and better align computational pathology approaches with infarct biology, several neuropathological tasks can be conceptually defined for computational analysis. These include temporal staging (acute, subacute, and chronic), identification of infarct core and necrotic tissue, approximate delineation of peri-infarct regions, detection of hemorrhagic transformation, characterization of reactive gliosis and microvascular injury [7], and identification of cavitation. However, many of these pathological features exhibit biologically continuous and spatially heterogeneous transitions, making precise lesion boundary definition challenging even for expert neuropathologists. These tasks, along with representative whole-slide image (WSI) annotation strategies and corresponding ML formulations, are summarized in Table 2.

**Table 2.** Infarct-specific pathology tasks, corresponding WSI annotation strategies, and machine learning formulations Table 2 long description.

Infarct pathology task	Representative histopathological features	Practical WSI annotation sources	Typical ML formulation
Temporal staging (acute, subacute, chronic)	Necrosis, macrophages, infiltration, reactive gliosis, tissue cavitation, vascular remodeling	Slide-level labels derived from neuropathology reports; expert staging annotations	Weakly supervised classification; MIL
Infarct core / necrosis identification	Eosinophilic neurons, tissue rarefaction, loss of architecture	Pixel-level or tile-level annotations by expert neuropathologists	Fully supervised segmentation (e.g., U-Net variants)
Penumbra surrogate regions	Selective neuronal loss, partial tissue injury	Region-level or coarse annotations; patch-level labels	Weakly supervised segmentation; MIL
Hemorrhagic transformation	Red blood cell extravasation, hemosiderin deposition	Pixel- or region-level annotations; semi-automated labeling	Detection and segmentation models
Microvascular injury	Arteriolosclerosis, vessel wall thickening, lumen narrowing	Vessel annotations; morphometric measurements	Object detection + quantitative morphometry
Cavitation (chronic infarct)	Tissue loss, cystic spaces	Slide-level labels or region-level annotations	Classification; segmentation

Here, annotation sources refer to practical strategies for generating training labels from WSIs, including expert annotations, slide-level diagnoses, and weak supervision; MIL = Multiple Instance Learning.

These task definitions span multiple spatial scales and annotation levels. Pixel-level annotations support supervised segmentation, while slide-level labels enable weakly supervised and multiple instance learning (MIL) approaches. Self-supervised pretraining further enables learning from large unlabeled datasets, highlighting the need for infarct-specific computational pathology frameworks.

## Machine learning methods for histological infarct analysis

Recent advances in computational pathology have demonstrated the feasibility of applying CNNs to WSIs for segmentation and quantification of tissue compartments, microvascular structures, and inflammatory responses in disease [48–52]. Encoder-decoder CNN architectures, patch-based learning, and attention-driven aggregation strategies have enabled scalable analysis of gigapixel images while preserving spatial context critical for neuropathological interpretation [47,50]. However, despite the use of similar architectures, *in vivo* neuroimaging has advanced through large multi-center datasets and standardized benchmarks, whereas digital pathology remains fragmented and dataset-limited, slowing methodological convergence [90,102], particularly for infarct-specific applications.

A recent comprehensive review by Rosado et al. examined the foundations, technological infrastructure, and emerging ML applications in digital neuropathology, with a primary focus on neurodegenerative diseases [102]. The authors highlight advances in WSI platforms, data management pipelines, annotation tools, and DL workflows, as well as persistent barriers related to data scale, interoperability, annotation burden, computational cost, and generalizability. Importantly, the review emphasizes the growing role of ML in quantitative tissue phenotyping, multi-modal data integration, and reproducible biomarker discovery, while noting that most current applications remain concentrated in Alzheimer disease and related dementias rather than cerebrovascular pathology [102].

Despite these emerging methodological opportunities, relatively few studies have developed infarct-specific computational pathology approaches. Existing work has largely emphasized tissue segmentation, or general small-vessel pathology rather than infarct-specific lesion detection or classification [50,74,94]. Advances in ML tools for digital pathology of brain tissue include BrainSec, an automated method for gray and white matter segmentation classification [50], and a combined workflow for amyloid-β plaque, a pathologic hallmark of Alzheimer disease, localization and quantification with expert-level accuracy [75]. Lou et al [94] developed ArtSeg-VasMorph, an end-to-end ML-based framework for automated detection, classification, and morphometric analysis of arteriolosclerotic vessels in the human brain on WSIs. Although these methods, to our knowledge, did not include cases of ischemic infarct, they establish transferable computational pathology paradigms, such as large-scale tissue segmentation, vascular morphometry, and reproducible patch aggregation, that can be directly applicable to infarct histological analysis. Only one study, to our knowledge, has utilized ML techniques to identify infarcts in brain histology slides [92]. Oliveira et al. provided a proof-of-concept pipeline for automated detection of microinfarcts and microhemorrhages in post-mortem brain tissue, demonstrating the feasibility of ML-based infarct screening in pathology [92].

While most existing approaches in digital pathology rely primarily on image-based features, these methods may not fully capture the broader clinical and contextual information associated with infarct pathology. Integrating complementary data sources, such as clinical or pathology report text, could offer an opportunity to enrich representation learning and enable more scalable and context-aware modeling. In this context, multimodal learning frameworks have emerged as a promising approach for combining heterogeneous data sources within a unified model [115].

As illustrated in Figure 4, multimodal learning frameworks move beyond simple parallel processing of image and text inputs by integrating representations within a shared embedding space, where explicit cross-modal interaction (e.g., cross-attention or feature-level fusion) enables joint representation learning. This allows the model to capture relationships between visual patterns and clinical context, rather than treating modalities as independent information streams.

**Figure 4.**
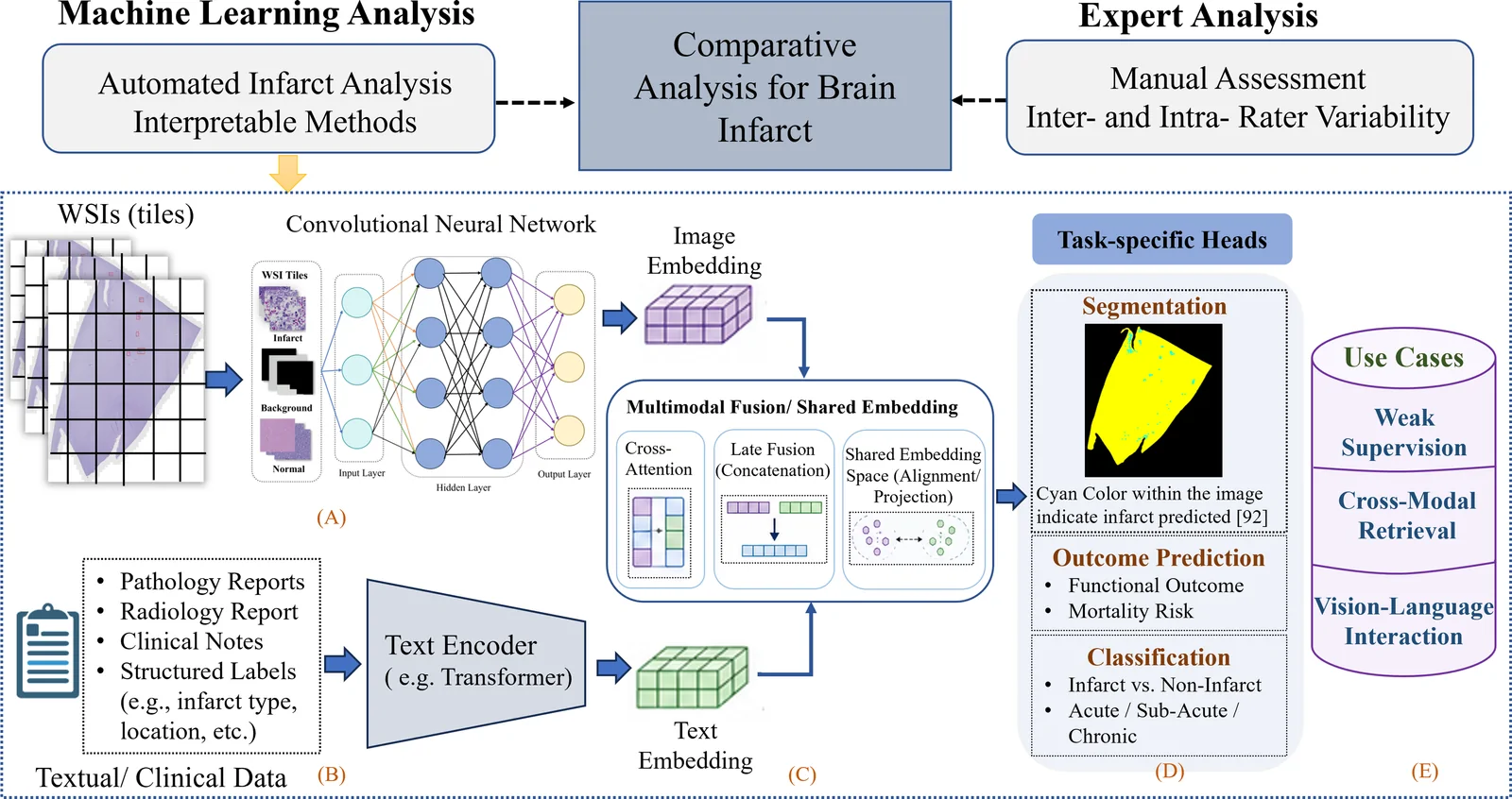
Multimodal learning workflow for cerebral infarct analysis using whole-slide images (WSIs) and clinical text. (A) WSIs are divided into image patches and processed through an image encoder to generate latent visual feature representations. (B) Clinical reports and associated textual data are processed using a transformer-based text encoder to generate contextual text embeddings. (C) Visual and textual embeddings are integrated within a shared multimodal embedding space through cross-modal interaction or feature-level fusion, enabling joint representation learning. (D) The fused representations support task-specific prediction heads for segmentation/infarct classification/clinical outcome prediction. Image-derived features typically dominate pixel-level tasks such as segmentation, while text-derived features provide complementary contextual supervision. (E) These multimodal frameworks additionally enable weak supervision, cross-modal retrieval of similar cases, and emerging vision-language applications for interactive interpretation and reporting.

In infarct analysis, image features typically remain the dominant signal for pixel-level tasks such as segmentation, while text may provide complementary contextual information. Accordingly, multimodal approaches are particularly well suited for applications such as weak supervision using text-derived labels, cross-modal retrieval of similar cases, and vision-language models that support interactive interpretation and reporting.

While similar architectural families, such as U-Net variants, attention mechanisms, and CNN–transformer hybrids, are increasingly adopted in both *in vivo* imaging and digital pathology, their roles differ substantially across these domains. In *in vivo* imaging, these architectures have supported spatially continuous lesion segmentation and outcome-oriented modeling, whereas in pathology, they have been primarily used for patch-level feature extraction and slide-level aggregation to manage gigapixel-scale images [64–66]. Differences in tissue sampling, spatial continuity, and availability of clinically aligned outcome labels may further limit direct transfer of *in vivo* imaging-based modeling strategies to pathology, contributing to non-parallel methodological evolution across domains.

Beyond task-specific pipelines, foundational DL strategies used in both digital pathology and *in vivo* imaging, including stain normalization, and self-supervised pretraining, offer important tools for addressing limited annotations and high intra-slide variability [51,81,90]. Emerging foundation and vision-language models such as CLIP [82], LLaVA-Med [79], and CONCH [77] further suggest opportunities for zero- and few-shot learning in settings where expert labeling is scarce [77,79,82]. Although related vision-language frameworks are beginning to be explored in *in vivo* imaging and digital pathology research, their application to stroke imaging and neuropathology remains limited due to the scarcity of paired image-text datasets and the substantial domain gap between natural images, radiologic scans, and cellular-scale histological patterns [67,103,104].

Overall, ML methods in digital pathology for cerebral infarcts evaluation remain a promising but underdeveloped research area, with opportunities to expand datasets for generalizability, improve harmonization strategies, and develop more standardized and clinically aligned task definitions

## Discussion, limitations, and future directions

*In vivo* imaging studies have demonstrated rapid methodological maturation, with increasingly sophisticated architectures (see Table 1). Challenges remain related to generalizability, interpretability, and clinical translation [33,40,59]. ML applications in digital pathology remain comparatively limited for stroke/infarcts; however, they offer unique opportunities to interrogate infarct pathophysiology at cellular and microvascular scales. Together, these domains illustrate complementary strengths and shared challenges that define the current state of AI-driven stroke research.

### Algorithmic Bias and Generalizability

Limited dataset diversity remains a fundamental barrier to generalizable ML-based infarct assessment. Most imaging models are trained on retrospective, single-center datasets with limited demographic representation and protocol variability, which can degrade performance when models are applied across institutions or scanner platforms [33,36,39]. These limitations increase the risk of algorithms performing well in controlled research environments but failing to maintain accuracy in real-world clinical settings.

Beyond technical variability, substantial epidemiological and neuropathological evidence demonstrates infarct burden, subtype distribution, and post-stroke outcomes can vary by age, sex at birth, and socio-cultural factors, where race and ethnicity are often used as proxies [13,54–57]. For example, women exhibit differences in gross infarct prevalence compared with men, while dementia cohorts reveal higher rates of macro- and microinfarcts among Hispanic and African American populations compared with non-Hispanic White individuals [55–57]. Training datasets that fail to reflect this heterogeneity risk encoding algorithmic bias and exacerbating gaps in stroke diagnosis and care.

In digital pathology, generalizability challenges are amplified by variability in staining protocols, tissue processing, and slide-scanning hardware, which introduce batch effects across institutions [58–62]. The scarcity of large, multi-center WSI datasets and limited access to expert-annotated infarct labels further restrict robust model validation and contribute to overfitting in narrowly defined tasks [13,64]. Improving demographic representation, cross-site validation, and data harmonization is therefore essential for equitable and clinically reliable deployment of ML in both imaging and pathology.

### Evaluation Frameworks and Benchmarking Limitations

Evaluation practices in stroke ML research remain highly heterogeneous, complicating method comparison and obscuring true clinical readiness. Differences in preprocessing pipelines, annotation conventions, and outcome definitions introduce substantial variability across reported results, while most studies rely on internal validation without independent external testing [40,63,92–94]. As a result, high-performance metrics may not translate into reliable performance in real-world settings.

Common metrics such as Dice coefficient and AUC provide useful aggregate measures but often fail to capture clinically relevant failure modes, including missed small infarcts, posterior fossa lesions, or early ischemic changes [32,34]. Moreover, these metrics rarely reflect downstream clinical consequences such as treatment decisions or functional outcomes. Importantly, clinical readiness cannot be assessed by accuracy metrics alone. Computational efficiency, inference time, hardware requirements, and robustness under real-time clinical constraints are rarely reported but critically influence feasibility in workflows. Models that require extensive preprocessing, large memory footprints, or prolonged inference may be impractical despite high reported accuracy.

In pathology, evaluation is further complicated by patch-based learning strategies, heterogeneous annotation standards, and limited access to slide-level ground truth, restricting reproducibility across laboratories [47,51,58–62].

Establishing multi-institutional benchmarks with harmonized annotation protocols, clinically meaningful endpoints, and prospective validation strategies will be critical for assessing true algorithmic progress and supporting regulatory and clinical adoption. Without such frameworks, methodological advances risk remaining confined to research settings.

### Interpretability and Clinical Trust

Clinical adoption of ML-based infarct assessment methods requires not only technical accuracy but also interpretability and trustworthiness. In *in vivo* imaging, interpretable model behavior can help clinicians assess whether predictions are driven by anatomically plausible features and identify potential failure cases. In digital pathology, interpretability can support quality control by highlighting histological regions influencing predictions and exposing systematic annotation inconsistencies. However, model reliability must ultimately be established through rigorous external validation and robustness testing across diverse datasets [33,35,59,60,72]. Practical deployment also requires attention to inference speed, data availability, integration with clinical information systems, and regulatory considerations, all of which remain underexplored in most ML stroke studies.

Bridging the gap between algorithm development and routine clinical use will benefit from interdisciplinary collaboration among persons across medical domains, including neurologists, radiologists, pathologists, data scientists, ML engineers, and regulatory bodies, alongside infrastructure that supports secure data sharing and continuous model monitoring.

### Future directions

Future progress in ML-based infarct analysis will depend on both architectural innovation and improvement in data diversity, validation rigor, and problem formulation. In *in vivo* imaging, incorporating longitudinal data and clinically meaningful endpoints may enhance the utility of AI systems beyond lesion detection toward prognosis and treatment planning [46,78–89]. However, achieving this requires harmonized, multi-institutional datasets with set parameters for acquisition and annotation protocols.

Emerging ML strategies, including explainable AI, semi-supervised learning, graph-based models, and domain adaptation, offer promising tools for improving robustness and reducing dependence on exhaustive expert labeling, particularly in data-limited pathology settings [64–69,74]. Model efficiency techniques, such as pruning and knowledge distillation, may further facilitate deployment in time-sensitive clinical environments by reducing computational burden [69].

Beyond algorithmic innovation, future progress will depend on coordinated advances in data infrastructure and multimodal research frameworks. Harmonized digital pathology pipelines, FAIR data principles [93], and shared neuropathology reference atlases are needed to support large-scale validation and benchmarking [102,108]. Integrating histology with spatial transcriptomics, proteomics, and clinical data may further enable biologically grounded characterization of infarct evolution beyond morphology alone [102]. Such multimodal approaches create opportunities to move beyond task-specific pathology models toward systematic mapping of ischemic microstructure, including neuronal loss, gliosis, inflammation, and microvascular remodeling across infarct and peri-infarct regions (see Table 2 for task examples). Federated and privacy-preserving learning frameworks additionally offer pathways for multi-institutional collaboration without centralized data sharing [102]. Importantly, methodological advances from *in vivo* imaging, such as multi-scale feature learning, attention mechanisms, domain adaptation, and uncertainty estimation, are directly transferable to digital pathology workflows and may accelerate scalable detection and quantification of microinfarcts and microvascular injury, strengthening biological interpretation of imaging biomarkers.

Furthermore, contextualizing digital pathology in parallel with multimodal clinical, radiological, and genetic data can provide a holistic view of brain infarcts [68–70]. For example, image registration enables alignment of *in vivo* radiological images with corresponding histological data, while ML algorithms automate multimodal feature extraction; however, translating these approaches into robust pipelines requires addressing several key technical challenges [70,71] (some highlighted in Figure 2). First, multi-scale (cross-scale) registration is inherently complex, as MRI operates at millimeter-scale resolution, whereas histological WSIs capture micrometer-scale tissue architecture, requiring hierarchical and deformable alignment strategies. Second, antemortem–postmortem differences introduce variability due to tissue shrinkage, deformation, and distortion during fixation and sectioning, complicating spatial correspondence. Third, domain adaptation across modalities remains an open challenge, as MRI and histology differ substantially in contrast mechanisms and feature representations, necessitating robust methods to bridge cross-modal domain shifts. Addressing these issues with advanced ML and data processing methods promises a more robust and generalizable framework for infarct detection and characterization.

## Conclusion

This review summarizes recent advances in DL and ML approaches for cerebral infarct analysis, highlighting the maturation of DL methods for *in vivo* imaging and the emerging yet underexplored role of digital pathology in stroke research. DL applied to CT and MRI has progressed from handcrafted ML techniques to sophisticated convolutional and hybrid architectures, achieving high accuracy in stroke detection, segmentation, and outcome prediction, with some being FDA approved beginning in 2018 [111]. This underscores the growing technical capabilities of AI in cerebrovascular imaging.

At the same time, persistent limitations in spatial resolution, generalizability, and reproducibility continue to restrict the clinical impact of *in vivo* imaging-based models, particularly for infarcts. These challenges emphasize architectural innovation alone is insufficient without parallel advances in data heterogeneity, harmonized evaluation, and external validation. In digital pathology, progress is similarly constrained by limited infarct-specific datasets, variability in tissue processing and staining, and the absence of harmonized benchmarks, highlighting the need for larger, multi-institutional cohorts and validated annotation standards.

By aligning technical innovation with improvements in data quality, validation rigor, and biological interpretability, future work can better leverage both *in vivo* imaging and digital pathology to deepen our understanding of cerebral infarction and support reliable and equitable applications of AI in stroke imaging and research.

## Supporting information

10.1017/cts.2026.10799.sm001 Chauhan et al. supplementary material Chauhan et al. supplementary material

## Data Availability

Not applicable.

## Supplementary material

The supplementary material for this article can be found at https://doi.org/10.1017/cts.2026.10799.

## Table 2. Long description

A table comparing infarct pathology tasks, representative histopathological features, practical WSI annotation sources, and typical ML formulation. The table has four columns: Infarct pathology task, Representative histopathological features, Practical WSI annotation sources, and Typical ML formulation. It contains seven rows, each detailing specific tasks and their corresponding features, annotation sources, and ML formulations. Row 1: Temporal staging (acute, subacute, chronic), Necrosis, macrophages, infiltration, reactive gliosis, tissue cavitation, vascular remodeling, Slide-level labels derived from neuropathology reports; expert staging annotations, Weakly supervised classification; MIL. Row 2: Infarct core / necrosis identification, Eosinophilic neurons, tissue rarefaction, loss of architecture, Pixel-level or tile-level annotations by expert neuropathologists, Fully supervised segmentation (e.g., U-Net variants). Row 3: Penumbra surrogate regions, Selective neuronal loss, partial tissue injury, Region-level or coarse annotations; patch-level labels, Weakly supervised segmentation; MIL. Row 4: Hemorrhagic transformation, RBC extravasation, hemosiderin deposition, Pixel- or region-level annotations; semi-automated labeling, Detection and segmentation models. Row 5: Microvascular injury, Arteriolosclerosis, vessel wall thickening, lumen narrowing, Vessel annotations; morphometric measurements, Object detection + quantitative morphometry. Row 6: Cavitation (chronic infarct), Tissue loss, cystic spaces, Slide-level labels or region-level annotations, Classification; segmentation.

Navigate back to Table 2..
