# Supplementary material for: Deep learning methods for the detection of stroke for neuropathology: Lessons learned from in vivo imaging to digital pathology
Source: J Clin Transl Sci. 2026 Jul 28;10(1):e166. doi: 10.1017/cts.2026.10799 (PMC13748995; doi:10.1017/cts.2026.10799)
Supplement: Supplementary file 1 [file S2059866126107997sup001.docx]

**Deep Learning Methods for Detection of Stroke for Neuropathology: Lessons learned from *In-Vivo* Imaging to Digital Pathology**

**Supplementary Material**

TABLE A1. Summary of selected studies focused on the age-based categorization of acute, subacute, and chronic infarcts.

| **Source** | **Infarct Definition based on Age** |
| --- | --- |
| Raynaud *et al*., 1989 [1] | *Categories considered: Subacute and Chronic (Modality- CT)*   - subacute period:3 -50 days - chronic period: > 3 months |
| Chuaqui *et al*., 1993 [2] | - ***first period 1-4 days*:** predominance of eosinophilic neurons and necrotic oligodendrocytes - ***second period 5-7 days****:* appearance of macrophages and newly formed blood vessels - ***third period 8-14 days***: neuronal ghosts, macrophages, astrocytic proliferation, including gemistocytes, and absence of neutrophils - ***fourth period 15-27 days****:* no identified eosinophilic neurons, nor necrotic oligodendrocytes nor myelin in the central portion of the infarct. |
| Román *et al.,* 1993 [3] | *Arterial territorial infarction.*   - ***Acute stage: up to 72 hours,*** defined area of coagulation necrosis with adjacent areas of incomplete ischemic injury. - ***Subacute stage: 3 days to 2 weeks****,* tissue destruction containing abundant neutrophils, lipid-laden macrophages, and peripheral astrocytic activation.   ***Chronic stage: >2 weeks,*** wholly or partly cavitary lesions with or without lipid-laden macrophages and with minimal or no evidence of hemosiderin deposition. |
| Gideon *et al*., 1994 [4] | *Categories considered: Acute and Chronic (Modality- MRI)*   - **acute stage**: < 2 days   **chronic stage**: >6 months |
| Mena *et al*., 2004 [5] | *categories considered: Acute and Chronic*  ***Acute neuronal injury: 1-2 days***   - Absence of: macrophages, gemistocytes, (neuroaxonal) spheroids, cavitations, neovascularization, perivascular cuffing, mononuclear cells - Presence of: eosinophilia, cytoplasmic vacuolation, shrinkage and ghost cells, spongiosis, coagulative necrosis, polymorphonuclear leukocytes   ***Acute inflammatory phase: 3-37 days***   - Absence of: polymorphonuclear leukocytes - Presence of: coagulative necrosis, neuronal changes, neuronal ferrugination, spongiosis, spheroids, inflammatory mononuclear cells, perivascular cuffing, macrophages, gemistocytes, neo-vascularization, cavitation   ***Chronic inflammatory phase: 10 days - 53 years***   - Absence of: polymorphonuclear leukocytes, neuronal changes, spongiosis, coagulative necrosis, gemistocytes, neovascularization - Presence of: neuronal ferrugination, spheroids, mononuclear inflammatory cells, perivascular cuffing, macrophages, cavitation   ***Resorption phase: 26 days – 23 years***   - Absence of: inflammation, neuronal changes, spongiosis, coagulative necrosis, gemistocytes, neovascularization - Presence/absence of: neuronal ferrugination, spheroids, macrophages, cavitation |
| Birenbaum *et al*., 2011 [6] | *Categories considered: Acute, Subacute, and Chronic (Modality- CT)*   - **acute**: less than 24 hours - **subacute**:24 hours to 5 days   **chronic**: weeks |

FIGURE A1. Examples of selected hallmarks of infarcts of different ages with the absence or presence of a hemorrhagic component in whole slide images (WSI) of hematoxylin and eosin-stained brain tissue; a) subacute to chronic infarcts show macrophages and reactive astrocytes near the infarct border (glial scar), whereas b) acute hemorrhagic infarcts show extravascular red blood cells with no evidence of reactive glia or macrophages.

**Macrophages**

FIGURE A2. Example of cystic infarct detected in whole slide images (WSI, center) of hematoxylin and eosin-stained brain tissue of the left occipital periventricular white matter. In the center image, there is a cystic infarct, characterized by increased “white spaces” resulting from tissue necrosis. Surrounding boxes to the left or right are zoomed images of selected areas of the infarct. Reactive astrocytes (black arrows in the magnified image on the right) are present at the margin, and scattered macrophages (red arrows in the magnified image on the right, and black arrows within the lower left image) are present. Also, there are hemosiderin, a breakdown product of hemoglobin, and hemosiderin-laden macrophages (upper left panel- black arrow).

**TABLE A2. Brief summary of** advantages and disadvantages of different imaging modalities for cerebral infarct assessment.

| **Neuroimaging Modality** | **Advantages** | **Disadvantages** |
| --- | --- | --- |
| Non-contrast CT | - Fast acquisition time - High spatial resolution - Highly sensitive detecting acute intracranial hemorrhage Less expensive than other imaging modalities - Widely available | - Exposure to ionizing radiation - Lower sensitivity for early ischemic infarction and small infarcts Lack of information about the underlying cause of stroke |
| CT Angiography | - Fast acquisition time - High spatial resolution - Highly sensitive in detecting stenosis or occlusion of blood vessels - Sensitive to the extent and severity of brain stroke - Provides information about the location of brain stroke | - Exposure to ionizing radiation - Potential renal toxicity of iodinated contrast agent - Contrast agents may cause allergic reactions - Less sensitive to stroke size |
| CT Perfusion | - Fast acquisition time - High spatial resolution - Sensitive to the extent and severity of brain infarction - Provides information on the underlying cause of the infarct | - Exposure to ionizing radiation - Potential renal toxicity of iodinated contrast agent - Contrast agents may cause allergic reactions |
| Diffusion-Weighted Magnetic Resonance Imaging | - High spatial resolution - Useful in the early diagnosis of brain infarction - Sensitive to the extent and severity of brain infarction - Provides information about the location of brain infarction - Does not use ionizing radiation, so it is considered safe imaging, including in pregnancy | - Long acquisition time - Sensitive to motion artifacts - Expensive - Not easily available in all healthcare settings - High false positives due to sensitivity to the change in brain tissues - Limited use in those with non-MRI compatible cardiac and other implanted devices |
| Magnetic Resonance Angiography | - Useful for high-resolution visualization of brain vessel anatomy - Does not use ionizing radiation, so it is considered safe imaging, including in pregnancy | - Long acquisition time - Sensitive to motion artifacts - Expensive - Not easily available in all healthcare settings - Overestimates stenosis - Limited use in those with non-MRI compatible cardiac and other implanted devices |
| Magnetic Resonance Perfusion | - Variable spatial resolution - Sensitive in detecting changes in blood flow in the brain - Provides information on the degree of tissue perfusion | - Long acquisition time - Sensitive to motion artifacts - Expensive - Not easily available in all healthcare settings - Potential renal toxicity of gadolinium-based contrast agent - Uses contrast agents that may cause allergic reactions - Limited use in those with non-MRI compatible cardiac and other implanted devices |
| Positron Emission Tomography (PET) | - Highly sensitive in detecting changes in metabolic activity and blood flow in the brain - Sensitive to the extent and severity of brain infarction - Provides information about the location of brain infarction - Quantifies physiological parameters and differentiates acute vs. chronic infarcts based on metabolic activity | - Long acquisition time - Sensitive to motion artifacts - Expensive - Not easily available in all healthcare settings - Uses of radioactive tracers with relatively short half-live, - Low spatial resolution |

**References:**

[1] Raynaud C, Rancurel G, Tzourio N, et al. SPECT analysis of recent cerebral infarction. *Stroke*. 1989;20(2):192-204. doi:10.1161/01.str.20.2.192
[2] Chuaqui R, Tapia J. Histologic assessment of the age of recent brain infarcts in man. *J Neuropathol Exp Neurol*. 1993;52(5):481-489. doi:10.1097/00005072-199309000-00006
[3] Román GC, Tatemichi TK, Erkinjuntti T, et al. Vascular dementia: diagnostic criteria for research studies. Report of the NINDS-AIREN International Workshop. *Neurology*. 1993;43(2):250-260. doi:10.1212/wnl.43.2.250
[4] Gideon P, Sperling B, Arlien-Søborg P, Olsen TS, Henriksen O. Long-term follow-up of cerebral infarction patients with proton magnetic resonance spectroscopy. *Stroke*. 1994;25(5):967-973. doi:10.1161/01.str.25.5.967
[5] Mena H, Cadavid D, Rushing EJ. Human cerebral infarct: a proposed histopathologic classification based on 137 cases. *Acta Neuropathol*. 2004;108(6):524-530. doi:10.1007/s00401-004-0918-z
[6] Birenbaum D, Bancroft LW, Felsberg GJ. Imaging in acute stroke. *West J Emerg Med*. 2011;12(1):67-76.
